# In vivo evaluation of a novel format of a bivalent HER3-targeting and albumin-binding therapeutic affibody construct

**DOI:** 10.1038/srep43118

**Published:** 2017-02-23

**Authors:** Tarek Z. Bass, Maria Rosestedt, Bogdan Mitran, Fredrik Y. Frejd, John Löfblom, Vladimir Tolmachev, Stefan Ståhl, Anna Orlova

**Affiliations:** 1 Division of Protein Technology, School of Biotechnology, KTH-Royal Institute of Technology, SE-106 91 Stockholm, Sweden; 2 Division of Molecular Imaging, Department of Medicinal Chemistry, Uppsala University, SE-751 83, Uppsala, Sweden; 3 Affibody AB, SE-171 63 Stockholm, Solna, Sweden; 4 Department of Immunology, Genetics and Pathology, Uppsala University, SE-75285, Uppsala, Sweden

## Abstract

Overexpression of human epidermal growth factor receptor 3 (HER3) is involved in resistance to several therapies for malignant tumours. Currently, several anti-HER3 monoclonal antibodies are under clinical development. We introduce an alternative approach to HER3-targeted therapy based on engineered scaffold proteins, i.e. affibody molecules. We designed a small construct (22.5 kDa, denoted 3A3), consisting of two high-affinity anti-HER3 affibody molecules flanking an albumin-binding domain ABD, which was introduced for prolonged residence in circulation. *In vitro*, 3A3 efficiently inhibited growth of HER3-expressing BxPC-3 cells. Biodistribution in mice was measured using 3A3 that was site-specifically labelled with ^111^In via a DOTA chelator. The residence time of ^111^In-DOTA-3A3 in blood was extended when compared with the monomeric affibody molecule. ^111^In-DOTA-3A3 accumulated specifically in HER3-expressing BxPC-3 xenografts in mice. However, ^111^In-DOTA-3A3 cleared more rapidly from blood than a size-matched control construct ^111^In-DOTA-TAT, most likely due to sequestering of 3A3 by mErbB3, the murine counterpart of HER3. Repeated dosing and increase of injected protein dose decreased uptake of ^111^In-DOTA-3A3 in mErbB3-expressing tissues. Encouragingly, growth of BxPC-3 xenografts in mice was delayed in an experimental (pilot-scale) therapy study using 3A3. We conclude that the 3A3 affibody format seems promising for treatment of HER3-overexpressing tumours.

The human epidermal growth factor receptor 3 (HER3) has in recent years been recognized as a key node in the complex signalling network of many different cancers, e.g. breast, ovarian, pancreas, prostate, and colon carcinomas. The human epidermal growth factor receptor (HER) family of receptor tyrosine kinases consists of four members: EGFR or HER1 (ErbB1), HER2 (ErbB2), HER3 (ErbB3) and HER4 (ErbB4). Binding of ligands to an extracellular receptor domain of these kinases induces receptor homo- or heterodimerization and activation of the intracellular tyrosine kinase domains, triggering downstream signalling cascades. HER3 binds two natural ligands, heregulin (or neuregulin 1) and neuregulin 2^1^. HER3 differs from the other receptor members in that it lacks a fully functional intracellular tyrosine kinase domain^2^. Upon ligand binding, HER3 heterodimerizes with other receptors of the HER family, forming a functional signalling unit. HER3 is implicated in de novo and acquired resistance against several targeted therapies, and is a major activator of the PI3K/Akt signalling pathway^3^. Consequently, the HER3 receptor has attracted attention as a key target for drug development^4^.

A number of monoclonal antibodies have been generated to HER3 and several of these are presently in clinical trials, e.g. seribantumab (MM-121)^5^, lumretuzumab (RG7116)^6^, elgemtumab (LJM716)^7^ and patritumab (U3-1287)^8^. In addition, a few bispecific immunoglobulin-based drug candidates have been investigated and entered clinical evaluations, e.g. duligotumab (MEHD7945A), a two-in-one symmetrical IgG molecule^9^, MM111, a fusion protein comprising two scFvs fused to human serum albumin (HSA)^10^ and istiratumab (MM-141), two scFvs fused to an IgG^11^.

In recent years, different so called non-immunoglobulin scaffold proteins have been developed as tumour targeting agents, mostly for medical imaging applications, but also for therapy purposes^12^,^13^. To our knowledge, the only reported alternative scaffold protein targeting HER3, is an affibody molecule^14^.

Affibody molecules^15^ constitute a type of alternative scaffold proteins that has demonstrated excellent properties for tumour targeting purposes^16^. They have a molecular weight around 6.5 kDa, forming a three-helix bundle fold with very fast folding kinetics and capacity to re-fold after denaturing^17^. These properties, along with a high solubility and high expression level in bacteria^16^ facilitates engineering of multimeric proteins such as bispecific or bivalent constructs^18^, which can be valuable for design of therapeutic constructs. Additionally, GMP-production of affibody-ABD fusion proteins is possible by prokaryotic cells, which makes it less expensive compared with the GMP-production of monoclonal antibodies^19^.

Affibody molecules have been investigated extensively as targeting probes for imaging of cancer^20^ and were found to be an attractive format as imaging agents. The small size ensures a rapid extravasation of affibody-based probes, efficient penetration in tumours and rapid renal excretion of unbound tracers^16^. High affinity generally provides high uptake and retention of affibody-based agents in tumours. A HER2-targeting affibody molecule^21^ has in fact proven to be a safe and efficacious radionuclide imaging agent in humans, and is currently in clinical development^22^,^23^.

The first generation anti-HER3 affibody molecule was selected using a combination of phage and bacterial display technology^14^ and was shown to compete with the binding of the natural ligand heregulin, and to bind human HER3 and murine ErbB3 of similar subnanomolar affinity, making potential preclinical studies in mice of relevance^14^. Interestingly, the anti-HER3 Z_HER3:05416_ and Z_HER3:05417_ affibody molecules suppressed heregulin-induced HER3 phosphorylation in different cell lines *in vitro*, and blocked heregulin-induced downstream signalling through the Ras-MAPK and the PI3K-Akt pathways^24^. In an *in vitro* proliferation assay, the first generation anti-HER3 affibody molecule demonstrated complete inhibition of heregulin-induced cancer cell growth^24^. The demonstrated anti-proliferative effect in two breast cancer cell lines encouraged further studies towards a HER3-targeted cancer therapy. For therapy applications based on blocking of receptor-ligand interaction, longer blood residence times are desirable. This can be readily achieved through genetic fusion to an albumin binding domain (ABD) that is 46 amino acid residues in size (MW = 5.2 kDa) and engineered to femtomolar affinity for human albumin^25^,^26^. This concept has been explored in preclinical affibody-based radiotherapy applications^27^, and would also be conceptually applicable in humans^28^.

Inspired by the vast number of emerging investigations based on bispecific targeting^29^, we designed a number of different bispecific, and bivalent affibody molecules targeting HER2 and HER3, which also included ABD, mentioned above, to allow extended circulation time in future *in vivo* studies^30^. We expected that some of the bispecific constructs targeting both HER2 and HER3 would demonstrate to be most potent, but surprisingly a bivalent HER3-targeting construct, with two HER3-specific affibody molecules flanking the ABD, Z_HER3:05417_ -ABD-Z_HER3:05417_, proved to be significantly more potent in blocking ligand-induced phosphorylation of the HER receptors, which suggests an anti-proliferative effect^30^. An affinity maturation of anti-HER3 affibody molecules was performed yielding the Z_HER3:08699_ affibody molecule with low picomolar affinity to HER3^31^. The new binders also demonstrated an improved thermal stability as well as complete refolding after denaturation. Moreover, inhibition of ligand-induced proliferation of HER3-positive breast cancer cells was improved substantially compared to the previously best-performing variant. In addition, radiolabeled affibody molecules showed specific targeting of a number of HER3-positive cell lines *in vitro* as well as efficient targeting of HER3 in *in vivo* mouse models, which indeed encourage future development towards HER3-targeted therapies^31^.

The aim of this study was to evaluate biodistribution and *in vivo* targeting properties of a potential therapeutic construct based on the affinity-matured anti-HER3 affibody molecule Z_HER3:08699_ and containing ABD for extension of residence time in circulation. A fusion construct containing two Z_HER3:08699_ building blocks connected by (S_4_G)_4_-linkers through an engineered ABD_035_ domain (Z_HER3:08699_-(S_4_G)_4_-ABD_035_-(S_4_G)_4_-Z_HER3:08699_, hereinafter denoted 3A3) was designed and produced in *E. coli*. As a control, a structurally identical construct containing affibody molecules binding to *Taq*-polymerase (Z_Taq_-(S_4_G)_4_-ABD_035_-(S_4_G)_4_-Z_Taq_, hereinafter denoted TAT) was generated. To study biodistribution, 3A3 and TAT variants with a site-specifically conjugated DOTA chelator derivative (designated as DOTA-3A3 and DOTA-TAT, respectively) were developed and labelled with the radionuclide ^111^In. We confirmed that the new high-affinity construct maintained the growth inhibitory effect in the HER3-expressing pancreatic cell line, BxPC-3. Biodistribution of the novel construct after administration using different routes was tested in tumour-free mice. Targeting specificity *in vivo* was confirmed using immunodeficient mice bearing HER3-expressing BxPC-3 xenografts. A pilot therapy study demonstrated that treatment with 3A3 caused significant growth delay of HER3-expressing BxPC-3 xenografts.

## Results and Discussion

### Design, production and *in vitro* characterization of bivalent affibody constructs

The constructs in the present study were designed as the bivalent affibody constructs studied previously^30^. In this format, two affibody units flank a 46 amino acid albumin binding domain (ABD_035_, hereinafter denoted ABD)^25^. In the current design, an affinity-matured HER3-binding affibody molecule Z_HER3:08699_^31^ replaced the earlier affibody version Z_HER3:05417_. The high-affinity HER3-binding affibody molecule is of interest since it was shown to efficiently inhibit ligand-induced proliferation of HER3-positive breast cancer cells *in vitro*^31^, and the bivalent format was chosen since it was the most potent in blocking ligand-induced phosphorylation of HER receptors in a comparison of different bispecific and bivalent constructs^30^. An albumin binding domain, ABD, engineered to femtomolar affinity for human serum albumin (HSA), which also binds serum albumins from other species with high affinity^25^, was expressed between the two affibody units, with the purpose of extending circulation times *in vivo*^26^. The bispecific construct is hence bivalent for HER3 and monovalent for albumin. A similar size-matched fusion protein TAT, binding the non-mammalian target *Taq* polymerase, was produced as a control protein.

The two bivalent fusion proteins were expressed in *E. coli*, recovered by ABD-affinity chromatography and reversed phase HPLC to a purity exceeding 98%, (Supplementary Fig. S1). The molecular masses as measured by mass spectrometry (Supplementary Fig. S1), 22,506 for 3A3 and 22,126 for TAT, were in a very good agreement with the expected theoretical values, 22,506 and 22,124, respectively.

**Figure 1:**
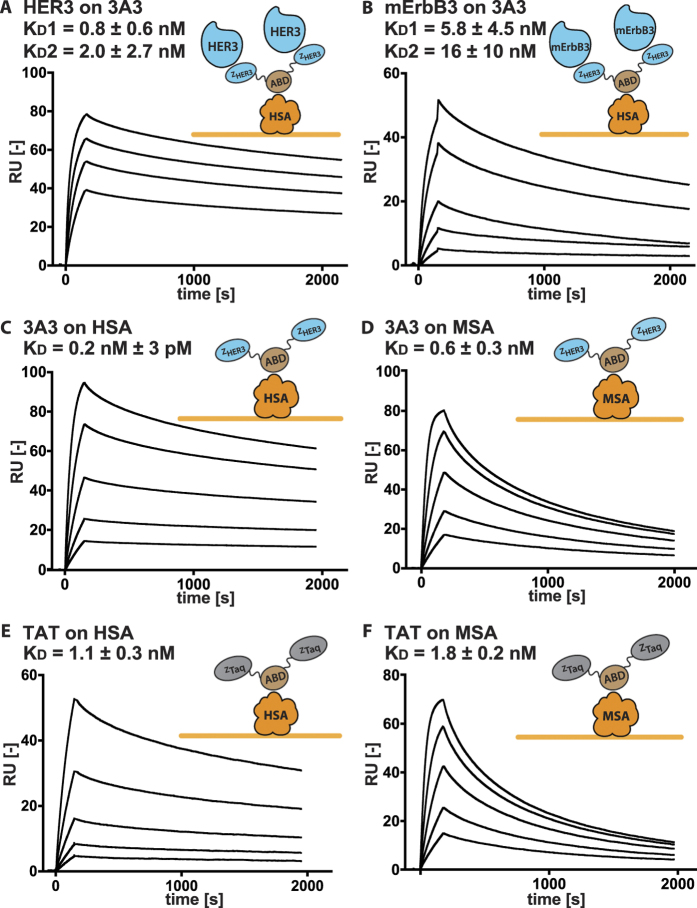
Results for the SPR affinity determinations. All analytes were stepwise diluted 1:2 and injected in series over: (**a**) immobilized HSA and 3A3 captured with subsequent injections of 15.6 to 125 nM of HER3. Please note that the dissociation between 3A3 and HSA has been subtracted, (**b**) immobilized HSA and 3A3 captured with subsequent injections of 20 to 500 nM of mErbB3. Please note that the dissociation between 3A3 and HSA has been subtracted, (**c**) immobilized HSA with subsequent injections of 1.6 to 25 nM of 3A3, (**d**) immobilized MSA with subsequent injections of 1.6 to 25 nM of 3A3, (**e**) immobilized HSA with subsequent injections of 1.6 to 25 nM of TAT, (**f**) immobilized MSA with subsequent injections of 3 to 50 nM of TAT.

High-affinity binding to HER3 and HSA was also verified using surface plasmon resonance (SPR). In the assay, 3A3 was captured onto immobilized human serum albumin, followed by analysis of binding to respective receptor in order to mimic the conditions in future *in vivo* experiments where 3A3 will be in complex with albumin when interacting with the receptor. The results from the experiment demonstrated slow dissociation for both targets (Fig. 1). The best fit was observed using a heterogeneous ligand-binding model. An explanation could be that the affinity for HER3 is influenced by the orientation of the affibody molecules, i.e. N- and C-terminal fusions affect the binding differently. Similar tendencies have been observed previously using fusions of other affibody molecules, such as the HER2-specific binder^32^. Assuming this binding mechanism, 3A3 has a distinct K_D_ value towards HER3 for each HER3-binding interaction due to its domain fusion character, 0.8 ± 0.6 nM and 2 ± 3 nM (Fig. 1). The size-matched control (TAT) did not bind to HER3 or mErbB3 as expected (Supplementary Fig. S2). The affinities for mErbB3 for 3A3 were 6 ± 4 nM and 16 ± 10 nM. Affinity for HSA was measured in the absence of HER3 and determined to 230 ± 3 pM for 3A3 and 1.1 ± 0.3 nM for TAT respectively. The affinity for MSA was nearly identical when compared to HSA (Fig. 1). The affinities of all three domains are likely influenced by the fusion and therefore lower than previously reported^25^.

To allow *in vivo* biodistribution studies with radiolabeled versions of the two bivalent affibody fusion constructs, variants were generated having a C-terminal cysteine residue, to allow DOTA-conjugation for radiolabeling. The DOTA-conjugated fusion proteins were re-purified by reversed phase HPLC to purities exceeding 98% (Fig. 1Sc-d). According to mass spectrometry analysis (Fig. 1Sc-d), the masses of the DOTA-conjugated constructs (23,079 for DOTA-3A3 and 22,755 for DOTA-TAT) were in excellent agreement with the expected theoretical values, 23,079 and 22,754, respectively. As for the non-labelled constructs, the mass differed a maximum of 1 Da from their theoretical mass, 3A3 with 22,506 Da theoretical and measured mass and TAT with 22,124 Da theoretical and 22,125 Da measured mass.

**Figure 2:**
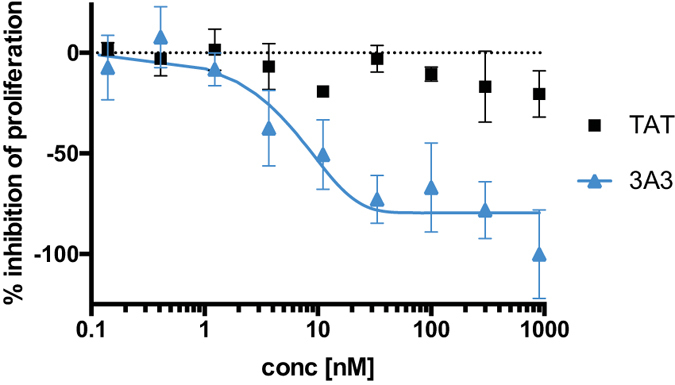
Cell proliferation was analysed by measuring cell growth inhibitory effects of the bivalent fusion proteins, 3A3 (filled triangles) and TAT (filled squares) incubated at concentrations ranging from 0.05 to 900 nM for 144 h with BxPC-3 cells. Cell viability was measured using a CCK8-kit. Maximum inhibition was set to the lowest absorbance signal of 3A3 treated cells.

A cell growth inhibition experiment was performed in order to investigate whether the treatment with new 3A3 bispecific affibody molecules would result in a decreased *in vitro* proliferation rate of BxPC-3, HER3-expressing pancreatic carcinoma cells. Cells were incubated for 6 days with different concentrations of 3A3 and TAT, ranging from 0.05 to 900 nM. Cell proliferation was measured by cell count-determination after 144 h using a CCK8 kit. BxPC-3 cells are autocrine for the HER3 ligand heregulin and absolute inhibition can therefore not be measured. The presence of 3A3 resulted in a significantly reduced cell proliferation, with a clear dose effect (Fig. 2), while increasing amounts of the control protein TAT had very limited effects on the cell proliferation.

### Radiolabeling, *in vitro* evaluation and biodistribution measurements of bivalent affibody constructs

To study the *in vivo* properties of the new construct, the affibody molecules were labelled with indium-111 with high yield (95 ± 6% for ^111^In-DOTA-3A3, n = 7; 99.8 ± 0.1% for ^111^In-DOTA-TAT, n = 2). After size-exclusion column purification, the compounds purities exceeded 99%. Label stability was tested in Phosphate-buffered saline (PBS) and under challenge with 500-fold molar excess of ethylenediaminetetraacetic acid (EDTA). After incubation during 90 min, 98 ± 2% (PBS) and 99 ± 1% (EDTA) of radioactivity was associated with ^111^In-DOTA-3A3. For ^111^In-DOTA-TAT, affibody-associated radioactivity was 99 ± 1% after incubation in both solutions.

**Figure 3:**
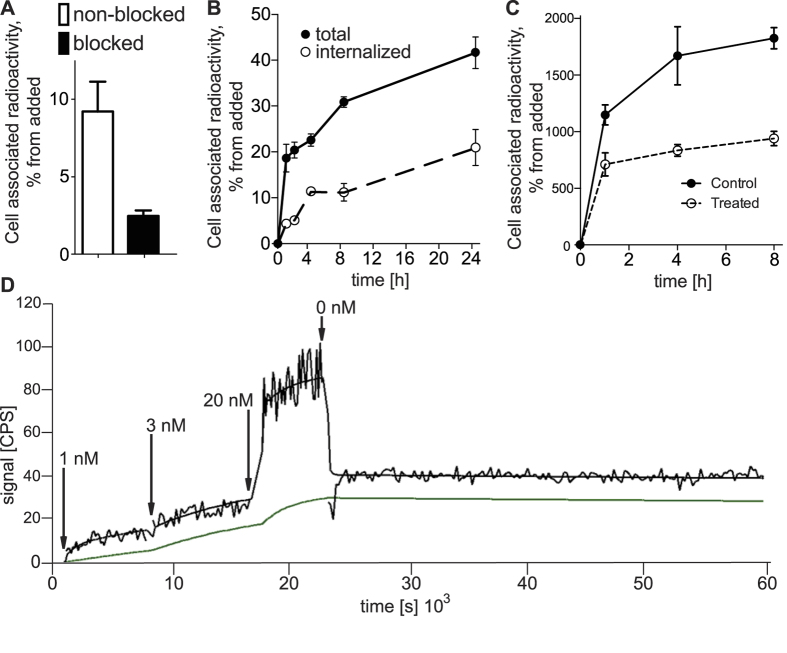
(**a**) Specificity of ^111^In-DOTA-3A3 binding to HER3-expressing BxPC-3 cells. Cells were incubated with 0.1 nM of ^111^In-DOTA-3A3, for saturation of receptors 5 nM solution of non-labelled 3A3 was used. (**b**) Uptake and internalization under continuous incubation with 0.1 nM of ^111^In-DOTA-3A3 with BxPC-3 cells at 37 °C. Data are presented as an average for 3 dishes ± SD. (**c**) Cell-associated radioactivity (cpm/10^6^ cells) during continuous incubation of BxPC-3 cells with ^111^In-DOTA-3A3 at 37 °C with and without 20 mM sodium azide/10 mM 2-deoxyglucose. (**d**) Representative uptake/retention LigandTracer sensorgrams of ^111^In-DOTA-3A3/HER3 interaction with BxPC-3 cells. Raw data (upper line) and data after subtraction of reference radioactivity (lower line).

An *in vitro* saturation test (Fig. 3a) demonstrated that the specific binding of ^111^In-DOTA-3A3 to HER3-expressing cells was preserved after labelling. Pre-incubation of cells with non-labelled affibody conjugate decreased significantly (p < 0.05) the cell-associated radioactivity, which demonstrated saturability of binding and suggested its specific character. Binding of ^111^In-DOTA-3A3 to HER3-expressing BxPC-3 cells was biphasic (Fig. 3b). A rapid binding phase during the first hour of incubation was followed by relatively slow increase of cell-associated radioactivity. Noticeable internalization of radioactivity was observed and already at 4 h about 50% of cell- associated radioactivity was internalized. To determine if the continuous increase of cell-bound activity was due to de novo formation of HER3, cells were incubated with ^111^In-DOTA-3A3 in the presence of sodium azide, which inhibits protein synthesis. In the case of sodium azide treatment, there was a rapid binding of ^111^In-DOTA-3A3 to BxPC-3 cells during the first 1 h of incubation, but no further increase of cell-associated radioactivity was observed (Fig. 3c). This suggests that new HER3 receptors are constantly formed by cancer cells and appear on the cellular membrane. In this way, HER3-expressed cells might process appreciable amount of bound 3A3.

Kinetics of binding to and dissociation from living BxPC-3 cells of ^111^In-3A3 was measured using LigandTracer Yellow at room temperature to exclude internalization (Fig. 3d). After subtracting background counts from sensorgrams, the best curve fitting was achieved by using a 1:1 model that is reasonable when taking into account the relatively low HER3 expression in BxPC-3 cells^33^. The on-rate was determined to around 1.6 ± 0.6 × 10^4^ (Ms)^−^1 and the off-rate to around 1.36 ± 0.09 × 10^−6^ s^−1^, resulting in an equilibrium dissociation constant of 0.10 ± 0.04^ × ^10^−9^ M. High affinity of the new 3A3 fusion protein to HER3 on cells was thus preserved after labelling.

Biodistribution of ^111^In-DOTA-3A3 was studied in NMRI mice with the aim to establish administration route, frequency and protein dose for planned *in vivo* therapy study.

**Figure 4:**
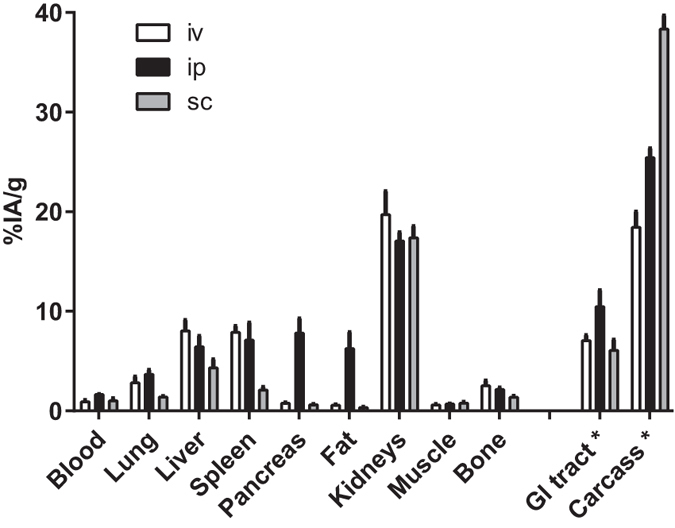
Biodistribution of ^111^In-DOTA-3A3 in NMRI mice at 24 h p.i. Compound was injected intravenously (iv), intraperitoneally (ip) or subcutaneously (sc). Results are presented as average of percent injected dose per gram tissue (%ID/g) for 4 animals ± SD. *Uptake in GI (gastrointestinal tract) and carcass is presented as %ID in whole sample.

Taking into account that a therapy study requires multiple injections, we reasoned that subcutaneous or intraperitoneal injections would be more suitable in mice than intravenous administrations. Biodistribution of ^111^In-DOTA-3A3 was measured at 24 h after intravenous, subcutaneous and intraperitoneal injections (Fig. 4 and Table 1). It was found that biodistribution of the radiolabeled conjugate was generally quite similar for all tested injection pathways except for in pancreas and abdominal fat. The biodistribution after intraperitoneal injection in the majority of tested organs and tissues had the closest resemblance with the biodistribution after intravenous injection, although blood concentration, and radioactivity retention in pancreas, abdominal fat, gastrointestinal tract and carcass were significantly (p < 0.05) higher for the intraperitoneal route. After subcutaneous injection, the ^111^In-DOTA-3A3 uptake was significantly (p < 0.05) lower than uptake after intravenous injection in lung, liver, spleen and bone, but higher in carcass. We believe that the elevated radioactivity uptake in pancreas after ip injection of ^111^In-3A3 was due to the lipophilic character of the conjugate and not receptor mediated. The conclusion is based on two observations: i) the very low pancreatic uptake (at the level of muscles) for other tested administration routes, as well as ii) the comparable radioactivity distribution in abdominal fat. It has to be noted that the weight of pancreas was in the range of 100–200 mg, and the total pancreatic uptake was 1–2% per whole tissue. Therefore, we selected intraperitoneal injection for further experiments.

Biodistribution of ^111^In-DOTA-3A3 at different time points was studied up to 168 h after intraperitoneal injection (Table 1). It was found that the compound was efficiently taken up into blood circulation after intraperitoneal injections. Radioactivity concentration in blood 6 h pi was 7.9 ± 0.7%ID/g that was comparable with radioactivity concentrations for ^177^Lu-ABD-Z_HER2:342_-Z _HER2:342_ (having two anti-HER2 affibody units fused at C-terminus of ABD) of 8 ± 1%ID/g at 8 h^27^ and for ^177^Lu-Z_HER2:2891_-ABD (having single anti-HER2 affibody unit fused at N-terminus of ABD) of 5 ± 1%ID/g at 4 h and 8 ± 2%ID/g at 24 h after subcutaneous injections^34^. The highest radioactivity concentration was found in kidneys that were identified as predominant excreting organ. Although radioactivity concentrations in blood at 6 h pi was similar to concentration of other ABD-fused affibody conjugates, clearance of radioactivity from blood was more rapid. At 24 h pi radioactivity concentration in blood was 1.64 ± 0.02%ID/g for ^111^In-3A3, which was more than 4-fold lower than for ABD-fused anti-HER2 conjugates mentioned above. On the other hand, the blood radioactivity concentration of ^111^In-DOTA-3A3 was much higher than the blood radioactivity concentration of ^111^In-labeled monomeric form of Z_HER3:08699_ non-fused with ABD (ca. 16-fold at 6 h and 7-fold at 24 h after injection)^35^. The results of this experiment demonstrated that the fusion with ABD enhanced residence time of 3A3 in circulation, but the magnitude of this enhancement was smaller than the effect for both monomeric and dimeric forms of anti-HER2 affibody molecules, fused at the N- or C-termini of ABD. There might be two reasons for this phenomenon: i) binding to albumin *in vivo* might be inferior for ABD flanked by affibody binders (e.g. due to steric hindrance) or ii) ^111^In-DOTA-3A3 might be efficiently sequestered from blood by mErbB3-expressing tissues.

**Figure 5:**
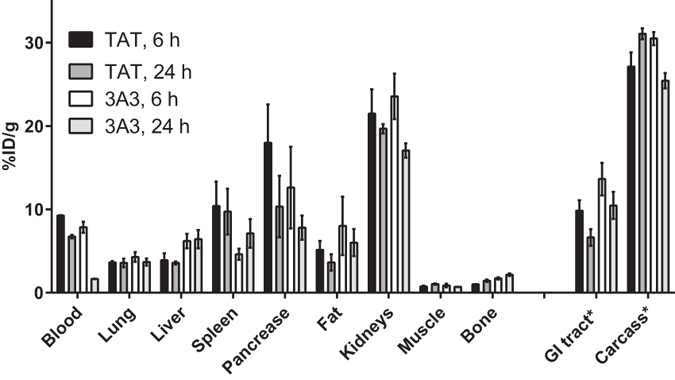
Comparison of biodistribution profiles of ^111^In-DOTA-3A3 and ^111^In-DOTA-TAT at 6 and 24 h after ip injections in NMRI mice. Results are presented as average of percent injected dose per gram tissue (%ID/g) for 4 animals ± SD. *Uptake in GI (gastrointestinal tract) and carcass is presented as %ID in whole sample.

We compared biodistribution of ^111^In-DOTA-3A3 and ^111^In-DOTA-TAT at 6 and 24 h pi to verify that ABD retained its ability to bind to albumin *in vivo* when placed between two affibody domains (Fig. 5 and Table 2S). The most pronounced difference in radioactivity concentrations was found in blood. At 6 h pi, radioactivity concentrations in blood were similar, but at 24 h pi the radioactivity concentration in blood for the ^111^In-DOTA-TAT (6.7 ± 0.2%ID/g) conjugate was 4-fold higher than for the ^111^In-DOTA-3A3 (1.64 ± 0.02%ID/g) conjugate. The blood concentration of ^111^In-DOTA-TAT was similar as the concentration of Z_HER2:2891_-ABD_035_-DOTA (8 ± 2%ID/g) in a murine model^34^. In addition, uptake in liver and gastrointestinal tract (i.e. in mErBb3-expressing organs) was significantly higher for the anti-HER3 conjugate. These data suggest that the ABD retained its binding to albumin and the rapid blood clearance of ^111^In-DOTA-3A3 conjugate could be attributed to its HER3/mErbB3 binding in normal tissues. Such interpretation is supported by *in vitro* data on strong affinity to the receptor and rapid internalization of the 3A3/receptor complex as well as data on preserved high binding affinities to HSA and MSA *in vitro*. The high hepatic uptake might raise the question if therapy using 3A3 would be safe. However, specific binding of a blocking therapeutic to normal tissues is not necessary harmful. For example, the high hepatic uptake of anti-HER3 affibody conjugate resembles that of anti-EGFR mAb 225, the murine predecessor of cetuximab^36^. In patients, 20–30% of the radiolabeled mAb was taken up by the liver already 1 h post injection via EGFR-mediated and dose-dependent mechanism^37^. Cetuximab is now approved for treatment of head-and-neck cancer and colorectal carcinoma^38^ with no reported side effects attributed to failure of liver function^39^.

The influence of injected protein dose on biodistribution was tested after injections of 40 and 160 μg of radiolabeled conjugate and additionally after repeated injection of 40 μg (40 μg of radiolabeled conjugate was injected at 24 h after injection of unlabelled conjugate). The results are presented in Table 1. The radioactivity uptake in mErbB3 expressing organs (lungs, liver, and organs of GI tract) was significantly higher after a single injection of 40 μg of protein, which suggests saturable character of the ^111^In-DOTA-3A3/mErbB3 interaction. We conclude that repeated injections of 3A3 or increasing injected dose would enable partial saturation of receptors in normal tissues. It has to be noted that partial saturation of mErbB3 in normal organs did not result in a significant increase of the blood concentration of ^111^In-DOTA-3A3, which might be expected. Some insight into this phenomenon might be gained by analysing binding and cellular processing of ^111^In-DOTA-3A3 (Fig. 3b and c). *In vitro* data suggest that an appreciable fraction of ^111^In-DOTA-3A3 that are bound to the receptor is internalized, and that formation of new receptors on the cell surface occurs continuously, which together enable internalization of a considerable amount of ^111^In-DOTA-3A3. It is reasonable to suppose that the pattern of processing would be the same for ErbB3 in healthy tissues, since processing of ErbB receptors is regulated by mechanisms preserved across species^40^. It is conceivable that mErbB3 provide still an efficient sequestering of ^111^In-DOTA-3A3 from blood even in the case of high receptor occupancy by non-labelled 3A3.

HER3 specific uptake of ^111^In-DOTA-3A3 in HER3-expressing BxPC-3 xenografts (12 ± 2 × 10^3^ receptors/cell^33^) was compared to uptake of control affibody conjugate ^111^In-DOTA-TAT and the results are presented in Table 2. Radioactivity uptake of anti-HER3 affibody conjugate in xenografts as well as tumour-to-blood and tumour-to-muscle ratios were significantly higher than uptake of the control conjugate (*p* < 3 × 10^−7^), despite of ca. 8-fold higher blood concentration of ^111^In-DOTA-TAT. This confirmed that ^111^In- DOTA-3A3 was capable of specific targeting of HER3-expressing tumours *in vivo*.

### *In vivo* therapeutic study of bivalent Affibody construct

**Figure 6:**
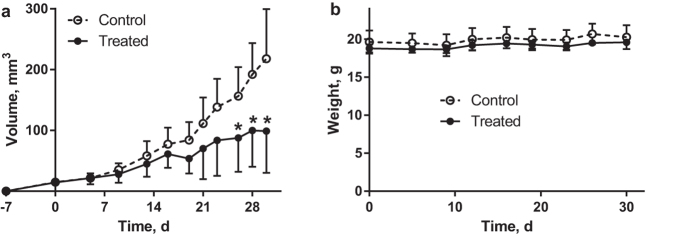
The results of anti-HER3 targeted therapy. (**a**) tumour volumes and (**b**) mice weights. Mice bearing BxPC-3 xenografts were intraperitoneally injected 3 times per week with 100 μl PBS with 80 μg of 3A3 (treated) or without it (control), n = 6. Mice were implanted at day -7 and treatment was started at day 0 and discontinued at day 30. At time points marked with *tumour volumes in control group were significantly bigger than in treated group (p < 0.05). Results are presented as average for 6 animals ± SD.

The pilot anti-HER3 *in vivo* therapy experiment was performed in mice bearing HER3-expressing BxPC-3 xenografts. Mice were injected intraperitoneally with 80 μg of 3A3 in PBS (treated group) or neat PBS (control group) three times per week during 4 weeks. Mice conditions were controlled according to Guidelines for Pain and Distress in Laboratory Animals from National Institute of Cancer (NIH, USA) adopted by Uppsala University; controlled parameters: exterior, general conditions, behaviour, stress, pain, ataxia, appetite, sores and blistering, skin colour, eye’s inflammation, porphyria, function of urinary and gastrointestinal systems, respiration, body-scoring, and body weight. None of these parameters were different between the treatment and control groups. Tumour size and animal weight were compared for treated and control groups and results are presented in Fig. 6. The repeated injections of anti-HER3 conjugate gave no visible side effects. The growth of xenografts in the treated group was slower than in the control group. Average tumour volume in the treated group became significantly smaller than in the control group from day 26 after therapy onset. Evidently, HER3-expressing xenografts responded to treatment using 3A3. The study was terminated at day 30 as the ethical permit was limited in terms of duration of treatment, number of injections and injected mass due to unknown toxicity profile of the first-in-class compound. However, even this pilot experiment suggests that treatment with 3A3 is well tolerated and slows growth of HER3-expressing xenografts.

### Concluding remarks

Treatment of metastatic cancer is challenging and therapeutic agents that specifically target (over)expressed receptors in tumours can significantly improve therapy outcome. Monoclonal antibodies are used for cancer therapy in clinics to interrupt ligand binding and, in this way, inhibit downstream proliferation-promoting signalling of such receptors as EGFR^41^ and VEGFR^42^. Two anti-HER3 monoclonal antibodies blocking heregulin binding to the receptor were reported in Phase I clinical trials, AV-203 (NCT01603979) and RG7116 (NCT01482377)^43^. Another approach is to lock the receptor in its inactive form, e.g. by binding domain II and IV^7^ or by promoting homodimerization^44^. These mechanisms of action can be achieved also with engineered scaffold proteins. For example, both HER3- and PDGFRβ-binding affibody molecules can block ligand-induced phosphorylation^24^,^45^.

The major issue with small scaffold proteins in therapy applications is their rapid renal clearance as they filter rapidly through glomerular membranes^20^. This complicates maintaining a sufficiently high concentration in blood and extracellular space. In addition, immunoglobulins are rescued from degradation by endothelial cells by interaction with neonatal Fc receptor (FcRn) and subsequent recycling^46^, while scaffold proteins usually lack this feature. Earlier, fusion with ABD has been successfully used to reduce renal reabsorption of affibody-based conjugates for radionuclide therapy^34^. This study provides evidence for that the ABD-fused affibody molecules can be successfully used *in vivo* for specific targeting and blocking of proliferation-driving receptors in malignant tumours. The fusion of affibody molecules with ABD provides a non-covalent binding to albumin, resulting in an adduct that is bigger than the cut-of size of glomerular filtration, ca. 60 kDa. Moreover, albumin is also recycled via FcRn^26^. Since ABD is not interfering with the FcRn interaction and retains the strong binding to albumin also at the slightly acidic pH in the endosomes, ABD-fused scaffold proteins obtains a further extension of the circulatory half-life by this mechanism^26^. Finally, albumin is accumulated in tumours because of enhanced permeability and retention effect (EPR-effect), further facilitating concentration of adduct in tumours^47^. Importantly, an adduct of 3A3 with albumin has a smaller size compared to bulky antibodies (molecular weight of 89 kDa vs 150 kDa), which favours more efficient extravasation and diffusion in extracellular space of tumours^48^,^49^.

This study provides evidence that the ABD-fused affibody molecules can be successfully used *in vivo* for specific targeting and blocking of proliferation-driving receptors in malignant tumours.

The novel high-affinity affibody-ABD fusion protein Z_HER3:08699_-(S_4_G)_4_-ABD_053_-(S_4_G)_4_-Z_HER3:08699_ (3A3) provides efficient inhibition of growth of HER3-expressing cancer cells *in vitro*. In a realistic murine model, Z_HER3:08699_-(S_4_G)_4_-ABD_053_-(S_4_G)_4_-Z_HER3:08699_ binds to mErbB3-expressing murine tissues. Nevertheless, it is capable of specific accumulation in HER3-expressing human tumour xenografts. Furthermore, a pilot *in vivo* therapy study demonstrated that Z_HER3:08699_-(S_4_G)_4_-ABD_053_-(S_4_G)_4_-Z_HER3:08699_ can delay growth of tumours *in vivo*.

## Materials and Methods

### Statistical analysis

Obtained values are presented as average with standard deviation if not stated otherwise. Data were assessed either by an unpaired, two-tailed t-test or by one-way ANOVA with Bonferroni correction for multiple comparisons using GraphPad Prism (version 6 for Windows GraphPad Software) in order to determine significant differences (p < 0.05).

### Design, production and characterization of the bivalent affibody fusion proteins, with and without DOTA-conjugation

Gene fragments encoding the affinity-matured HER3-binding affibody molecule Z_HER3:08699_^31^, were introduced to replace the previous HER-binding affibody molecule, to yield an expression vector based on pET26B(+) (Novagen, Madison, WI) that encodes a tripartite fusion protein Z_HER3:08699_-(S_4_G)_4_-ABD_035_-(S_4_G)_4_-Z_HER3:08699_, (22.5 kDa) hereinafter denoted 3A3. DNA sequence verification was performed using BigDye Thermo Cycle Sequencing reactions with an ABI Prism 3700 instrument (Applied Biosystems, Foster City, CA). The same non-binding size-matched construct as previously used Z_Taq_-(S_4_G)_4_-ABD-(S_4_G)_4_-Z_Taq_ (denoted TAT)^30^ was included as control. For the *in vivo* biodistribution studies the two gene constructs were altered using Quickchange primers (Eurofins Genomics, Ebersberg, Germany) to introduce a C-terminal cysteine residue to each binder as a handle to introduce a DOTA chelator (Sigma, Macrocyclics, Dallas, TX).

The bivalent fusion proteins were produced in *E. coli* BL21*DE3 cells (Novagen) and purified using affinity chromatography on an anti-ABD Sepharose matrix (Affibody AB, Solna, Sweden) The proteins were loaded on the column in TST running buffer (25 mM Tris-HCl (Sigma), 1 mM EDTA (VWR, Radnor, PA), 200 mM NaCl (Honeywell, Morris Plains, NJ), 0.05% Tween 20 (VWR)) pH 8, washed with ammonium acetate (VWR) pH 5.5 and eluted with 0.1 M acidic acid (VWR) at pH 2.9, then freeze dried overnight. As a second purification step a 1200 series HPLC system and a Zorbax C18 semi-preparative column (Agilent Technologies, Santa Clara, CA) was applied. Here, the fusion proteins were suspended in water with 0.1% trifluoroacetic acid (Merck, Darmstadt, Germany) and loaded onto the column then eluted via a gradient increase of acetonitrile (Merck) in the buffer (Fig. 1S). The molecular masses of the purified proteins were confirmed using a 6520 Accurate-Mass Q-TOF LC/MS (Agilent Technologies). The purity of the proteins was determined using an analytical column Zorbax 300B-C18 (Agilent Technologies) on 1200 series RP-HPLC (Agilent Technologies). Retained affinity to His6-ErbB3 (Sino Biological, Beijing, China) was verified on a BiaCore T200 system (GE Healthcare, Uppsala, Sweden) using a CM5 sensor chip with immobilized murine and human serum albumin (both Sigma-Aldrich, Darmstadt, Germany) according to previously published protocol^30^. Both bivalent binders, 3A3 and TAT were captured on the surface at 6 nM for 12 s to allow the ABD to bind to the immobilized surface with both target binding affibody moieties available for interaction. After the capture step dilutions of murine ErbB3 and human HER3 were injected at concentrations between 0.5 and 500 nM for 150 s for association and 1800 s for dissociation. 3A3 and TAT were injected at concentrations between 1 and 50 nM. Flow rates were kept at 10 μl/min with phosphate-buffered saline supplemented with 0.1% Tween 20 (PBST) as running buffer in all experiments and 10 mM hydrochloric acid to regenerate the surface. A 1:1 binding model was used to determine the affinity to albumin. A model allowing for a heterogeneous ligand was used to fit data to determine the affinities towards murine and human ErbB3/HER3 (Fig. 1).

Conjugation of the maleimide derivative of DOTA chelator to the constructs with a C-terminal cysteine residue was done according to a method published earlier^50^. Subsequently, the DOTA-conjugated fusion proteins were re-purified by RP-HPLC and the molecular masses were confirmed LC/MS as described above.

### Cell proliferation assay

Cells for *in vitro* and *in vivo* experiments were purchased from American Type Tissue Culture Collection (ATCC, Manassas, VI). The cell line BxPC-3 (pancreatic carcinoma) was cultured in RPMI media (Life Technologies, Carlsbad, CA) supplemented with 10% fetal bovine serum (FBS, Life Technologies) and 1% Penicillin/Streptomycin (PEST) and grown in incubator at 37 °C and 5% CO_2_. Trypsin-EDTA (0.25% trypsin, 0.02% EDTA in buffer) (Biochrom AG, Berlin, Germany) was used to detach cells.

For use in *in vitro* proliferation assays cells were passaged to RPMI medium supplemented with 2% dialyzed FBS at a density of 4000 cells per well in 96-well plates with three replicates per construct and in two independent experiments. Cells were incubated for 6 days with 0.05 to 900 nM of either bivalent affibody construct or no construct. BxPC-3 cells are autocrine for heregulin, which was therefore not added to the cells. Cell survival was measured by adding 10 μl of cell counting kit 8 (CCK8) solution (Sigma-Aldrich, St. Louis, MO) and further incubated at 37 °C for 6 h before measuring the optical density at 450 nm with a Tecan Sunrise microplate reader (Tecan Group, Männedorf, Switzerland). OD measurements were collected and adjusted to the maximal OD reached by wells containing no affibody constructs.

### Labelling of DOTA-3A3 and DOTA-TAT with ^111^In

^111^In-indium chloride was purchased from Covidien (Minneapolis, MN). High-quality Milli-Q water (resistance higher than 18 MΩ/cm) was used for preparing solutions. Buffers for labelling were purified from metal contamination by Chelex 100 resin (Bio-Rad Laboratories, Hercules, CA). The purity of labelled affibody molecule was verified by radio instant thin-layer chromatography (ITLC, 150–771 DARK GREEN, Tec-Control Chromatography strips from Biodex Medical Systems, New York, NY). The distribution of radioactivity along the chromatography strips was measured by a Cyclone^TM^ Storage Phosphor System using the image analysis software OptiQuant^TM^ (PerkinElmer, Waltham, MA).

For labelling, affibody conjugate (40 μg, 1 mg/mL in PBS) was mixed with 60 μL 0.2 M ammonium acetate buffer (acetic acid/ammonium hydroxide), pH 5.5, and 20 μL ^111^In-chloride solution (15 MBq). The mixture was incubated at 85 °C for 40 min. Then reaction mixture was analysed by radio-ITLC eluted with 0.2 M citric acid, pH 2.0. In this system, radiolabeled affibody molecules remain at the application point (R_f_ = 0) while free ^111^In or ^111^In-chelates migrate with the solvent front (R_f_ = 1). The analytical system was verified using a blank experiment, where no affibody molecule was added to the reaction mixture (less than 0.5% of radioactivity remained at the application point). To ensure high radiochemical purity, the conjugates were purified using disposable NAP-5 columns (GE Healthcare) according to the manufacturer’s instructions.

To check labelling stability, ^111^In-DOTA-3A3 and ^111^In-DOTA-TAT were incubated in 500-fold molar excess of EDTA at room temperature and affibody-bound radioactivity was measured using radio-ITLC. Control samples were incubated in PBS. Experiment was performed in duplicates.

### *In vitro* experiments

*In vitro* studies were performed according to the methods described earlier^51^. All *in vitro* studies were performed in triplicates.

The specificity of ^111^In-DOTA-3A3 binding to HER3-expressing cells was evaluated using the BxPC-3 cell line. For an *in vitro* specificity test a solution of radiolabeled affibody molecule (0.1 nM) was added to cell plates (10^6^ cells/dish). For blocking, 5 nM of non-labelled affibody molecule was added 15 min before radiolabeled conjugate. The cells were incubated for 1 hour at 37 °C. Thereafter, radioactivity was measured in cells and media to enable calculation of the fraction of cell-bound radioactivity. Radioactivity of samples was measured using automated gamma-spectrometer (1480 WIZARD, Wallac Oy, Finland).

Cellular processing (radioactivity uptake and internalization rate) was studied using BxPC-3 cells. Cells were (10^6^ cells/dish) incubated with labelled conjugate (0.1 nM) at 37 °C. At predetermined time points, media from a set of three dishes was removed, and the cells were treated with 0.2 M Glycine buffer containing 0.15 M NaCl and 4 M Urea pH 2.5, for 5 minutes on ice, in order to detach the membrane-bound radioactivity. To collect internalized radioactivity, the cells were treated with 1 M NaOH and incubated at 37 °C for 30 min. Samples were measured on radioactivity content and the percentage of cell associated and internalized radioactivity was calculated for each time point.

Additionally, BxPC-3 cells were incubated with ^111^In-DOTA-3A3 (0.1 nM) at 37 °C in presence of 20 mM sodium azide/10 mM 2-deoxyglucose at 37 °C for 8 h. At pre-determined time points, the medium from a set of three dishes was removed, the cells were detached by trypsin-EDTA solution, re-suspended and the radioactivity in cells was measured. Fraction of cell suspension was used for cell counting.

The binding of ^111^In-DOTA-3A3 to HER3-expressing cells BxPC-3 was measured in real time at room temperature using a LigandTracer Yellow instrument (Ridgeview Instruments AB, Vänge, Sweden) according to protocol described earlier^52^. The binding kinetics was measured at room temperature in triplicate for approximately 6.5 h using 1, 3 and 20 nM concentrations of ^111^In-DOTA-3A3. The retention measurements were at least 10 h. Interaction analysis and calculation of equilibrium dissociation constants (K_D_) were performed using TraceDrawer software (Ridgeview Instruments AB).

### *In vivo* experiments

All animal experiments were planned and performed in accordance with national legislation on laboratory animals’ protection and were approved by the Ethics Committee for Animal Research in Uppsala, Sweden. An average animal weight at the time of experiment was 26 ± 2 g for NMRI and 20 ± 1 g for Balb/c nu/nu mice, an average tumour size was 0.15 ± 0.08 g.

### Biodistribution studies

Mice (n = 4) were injected with radiolabeled affibody molecule in 100 μl of PBS (30 kBq/animal). The injected protein dose per animal was adjusted by non-labelled affibody conjugate. The animals were sacrificed at predetermined time points post injection (pi) by injection of a lethal dose of anaesthesia (20 μl of Ketalar/Rompun mixture per gram body weight: Ketalar (50 mg/ml, Pfizer), 10 mg/ml; Rompun, (20 mg/ml, Bayer, Shawnee, KS) followed by heart puncture and exsanguination with a syringe rinsed with heparin (5000 IE/ml, Leo Pharma, Copenhagen, Denmark). Tumours (when relevant) and samples of blood, lung, liver, spleen, stomach, small intestines, pancreas, abdominal fat, kidneys, salivary gland, muscle and bone were collected, weighed and their radioactivity was measured together with standard sample with radioactivity equal to injected. Tissue uptake was calculated as %ID/g. Radioactivity in carcass and gastrointestinal tract was calculated as %ID per whole sample.

To select the most suitable administration route, a biodistribution of ^111^In-DOTA-3A3 after intravenous, subcutaneous, and intraperitoneal injection was compared in NMRI mice. Mice were injected with 40 μg of ^111^In-DOTA-3A3 and the biodistribution was measured at 24 h pi.

Further, the biodistribution of ^111^In-DOTA-3A3 was measured in NMRI mice at 6, 24, 48, 72 and 168 h after intraperitoneal injection of 40 μg of radiolabeled affibody molecule. To evaluate influence of ErbB3-mediated uptake in normal tissues on clearance rate of ^111^In-DOTA-3A3, the biodistribution of ^111^In-DOTA-3A3and ^111^In-DOTA-TAT was compared at 6 and 24 h after intraperitoneal injections of 40 μg of radiolabeled affibody molecules. To evaluate influence of injected protein dose, the radioactivity biodistribution at 24 h after intraperitoneal injection of 40 and 160 μg of radiolabeled affibody molecule, as well as 40 μg of ^111^In-DOTA-3A3 affibody molecule injected 24 h after injection of 40 μg of unlabelled 3A3, was compared.

### *In vivo* specificity of HER3 targeting

To evaluate *in vivo* specificity of HER3 targeting, 40 μg of ^111^In-DOTA-3A3 or ^111^In-DOTA- TAT were intravenously injected in Balb/c nu/nu mice bearing BxPC-3 xenografts (subcutaneous inoculation of 5 × 10^6^ cells/100 μL PBS, 3 weeks before experiment). Biodistribution of both variants was measured at 24 h pi.

### Experimental *in vivo* therapy

To evaluate feasibility of HER3-targeting therapy and to estimate potential toxicity of the new affibody conjugate *in vivo*, a pilot experiment was performed. Since this was a first-in-class compound with unknown toxicity profile, the ethical permission was limited to 30 days of treatment, 3 intraperitoneal injections of 80 μg of 3A3 in PBS per week. Two groups (n = 6) of Balb/c nu/nu mice were subcutaneously implanted with 5 × 10^6^ cells BxPC-3 per animal. The treatment was started 7 days after tumour inoculation. The control group was treated with equal volume of PBS. Every mouse got 12 injections in total. Mice were weighed and xenografts were measured using callipers twice per week. The tumour volume was calculated using ellipsoid formula. Guidelines for Pain and Distress in Laboratory Animals from National Institute of Cancer (NIH, USA) were used for visual evaluation of animal conditions. The animals should be terminated in the case of tumour volume exceeding 1 cm^3^, open wound on tumour xenografts, 15% weight loss or 10% weight loss within one week.

## 

## Additional Information

**How to cite this article:** Bass, T. Z. *et al*. In vivo evaluation of a novel format of a bivalent HER3-targeting and albumin-binding therapeutic affibody construct. *Sci. Rep.* **7**, 43118; doi: 10.1038/srep43118 (2017).

**Publisher's note:** Springer Nature remains neutral with regard to jurisdictional claims in published maps and institutional affiliations.

## Supplementary Material

Supplementary Material

## Figures and Tables

**Table 1:** Biodistribution of radioactivity in NMRI mice after ip injections of ^111^In-DOTA-3A3 (40 μg and 160 μg – single injection, 40 μg repeated – 40 μg of non-labelled 3A3 were injected ip 24 h before ^111^In-DOTA-3A3).

	6 h*	24 h*	48 h*	72 h*	168 h*	24 h**	
	40 μg					40 μg repeated	160 μg
Blood	7.9 ± 0.7	1.64 ± 0.02^a^	0.32 ± 0.04^a^	0.11 ± 0.01^a^	0.022 ± 0.010^a^	1.4 ± 0.2	1.8 ± 0.3
Lung	4.3 ± 0.6	3.7 ± 0.4	1.9 ± 0.2^a^	1.2 ± 0.1^a^	0.27 ± 0.04^a^	1.4 ± 0.3^c^	1.6 ± 0.2^c^
Liver	6.2 ± 0.9	6 ± 1	5.37 ± 0.09	4.58 ± 0.08^a^	1.07 ± 0.05^a^	2.7 ± 0.5^c^	3.1 ± 0.6^c^
Spleen	4.6 ± 0.7	7 ± 2	7.0 ± 0.6^a^	6 ± 2^a^	4 ± 1^a^	1.63 ± 0.03^c^	1.7 ± 0.4^c^
Kidneys	24 ± 3	17.1 ± 0.9	13.4 ± 0.5^a^	10 ± 1^a^	2.5 ± 0.2^a^	38 ± 5^d^	42 ± 5^d^
Muscle	0.9 ± 0.2	0.68 ± 0.03^a^	0.42 ± 0.03^a^	0.31 ± 0.03^a^	0.10 ± 0.02^a^	0.40 ± 0.04^c,e^	0.49 ± 0.03^c^
Bone	1.7 ± 0.1	2.2 ± 0.2^b^	1.58 ± 0.06	1.1 ± 0.3	0.49 ± 0.05	0.8 ± 0.1^c^	1.0 ± 0.2^c^
GI tract^***^	14 ± 2	10 ± 2^a^	6.8 ± 0.6^a^	4.8 ± 0.4^a^	1.46 ± 0.09^a^	3.9 ± 0.4^c^	2.0 ± 0.1^c^
Carcass^***^	30.5 ± 0.8	25.4 ± 0.9^a^	18 ± 1^a^	12.4 ± 0.5^a^	4.0 ± 0.2^a^	35 ± 3^d^	34.8 ± 1.0^d^

Results are presented as average of percent injected dose per gram tissue (%ID/g) for 4 animals ± SD. *Data were assessed by an unpaired, two-tailed t-test in order to determine significant differences (p < 0.05). **Data were analysed together with data for 24 h using one-way ANOVA with Bonferroni correction for multiple comparisons (family-wise significance 0.05). ***Values for GI (gastrointestinal) tract and carcass are given as %ID per whole sample. ^a^Value is significantly lower than for the previous time point. ^b^Value is significantly higher than for the previous time point. ^c^Value is significantly lower than for 24 h pi of single dose of 40 μg (at least **P ≤ 0.01). ^d^Value is significantly higher than for 24 h pi of single dose of 40 μg (at least **P ≤ 0.01) e - value is significantly lower than for 24 h pi of single dose of 160 μg (*P ≤ 0.01).

**Table 2:** Biodistribution of ^111^In-DOTA-3A3 and ^111^In-DOTA-TAT in Balb/c nu/nu mice bearing BxPC-3 xenografts 24 h after iv injections with 40 μg of conjugates.

	Radioactivity uptake	
	^111^In-DOTA-3A3	^111^In-DOTA-TAT
Blood	0.6 ± 0.2	4.6 ± 0.3^a^
Salivary glands	3.0 ± 0.2^a^	1.5 ± 0.2
Lung	1.7 ± 0.2	2.6 ± 0.2^a^
Liver	14.6 ± 1.0	18.5 ± 0.8^a^
Spleen	4 ± 2	14 ± 3^a^
Stomach	1.2 ± 0.5^a^	0.59 ± 0.05
Small intestine wall	6 ± 1^a^	0.8 ± 0.1
Kidney	30 ± 4^a^	20 ± 2
Tumour	5.3 ± 0.7^a^	2.4 ± 0.2
Muscle	0.5 ± 0.1	0.50 ± 0.04
Bone	1.2 ± 0.3^a^	0.86 ± 0.10
GI^b^	5.8 ± 0.3^a^	1.4 ± 0.3
Carcass^b^	16.6 ± 0.3^a^	13 ± 1
	**Tumour to organ ratio**	
Tumour/blood	10 ± 2^a^	0.52 ± 0.05
Tumour/muscle	12 ± 3^a^	4.9 ± 0.5

Results for radioactivity uptake in tissues are presented as average of percent injected dose per gram tissue (%ID/g) for 4 animals ± SD. Data were assessed by an unpaired, two-tailed t-test in order to determine significant differences (P < 0.05). ^a^Value is significantly higher ^b^Values for GI (gastrointestinal) tract and carcass are given as %ID per whole sample.
