# Supplementary material for: In vivo evaluation of a novel format of a bivalent HER3-targeting and albumin-binding therapeutic affibody construct
Source: Sci Rep. 2017 Feb 23;7:43118. doi: 10.1038/srep43118 (PMC5322329; doi:10.1038/srep43118)
Supplement: Supplementary Material [file srep43118-s1.pdf]

In vivo evaluation of a novel format of a bivalent HER3-targeting and albumin-binding therapeutic affibody construct

Tarek Z. Bass, Maria Rosestedt, Bogdan Mitran, Fredrik Y. Frejd, John Löfblom, Vladimir Tolmachev, Stefan Ståhl and Anna Orlova

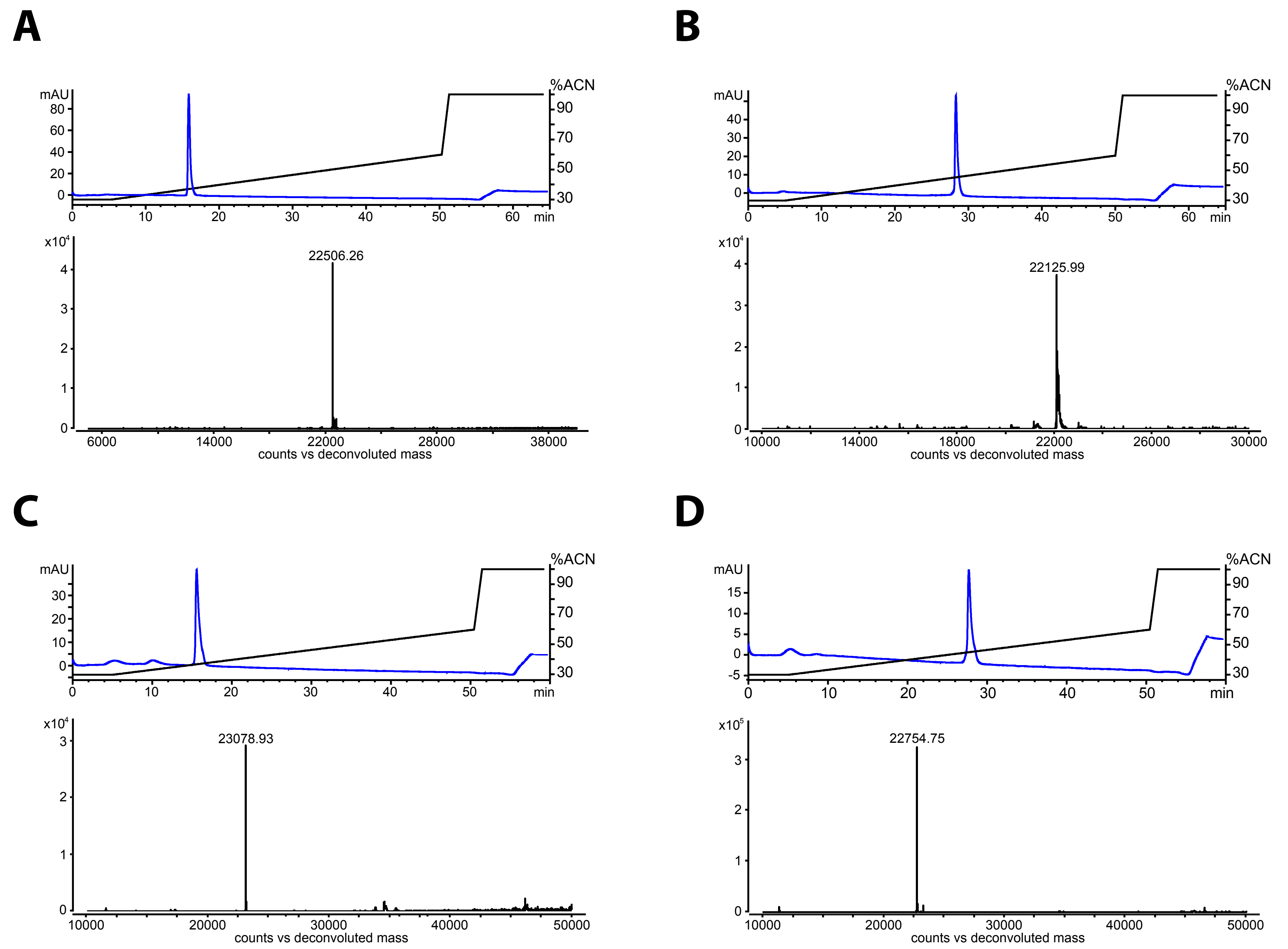

Figure 1S. HPLC chromatograms (top of each panel) and ESI-MS spectra (bottom of each panel) for (**a**) 3A3, (**b**) TAT, (**c**) DOTA-3A3 and (**d**) DOTA-TAT, demonstrated that all purities exceeded 98%, and confirmed the expected molecular masses.

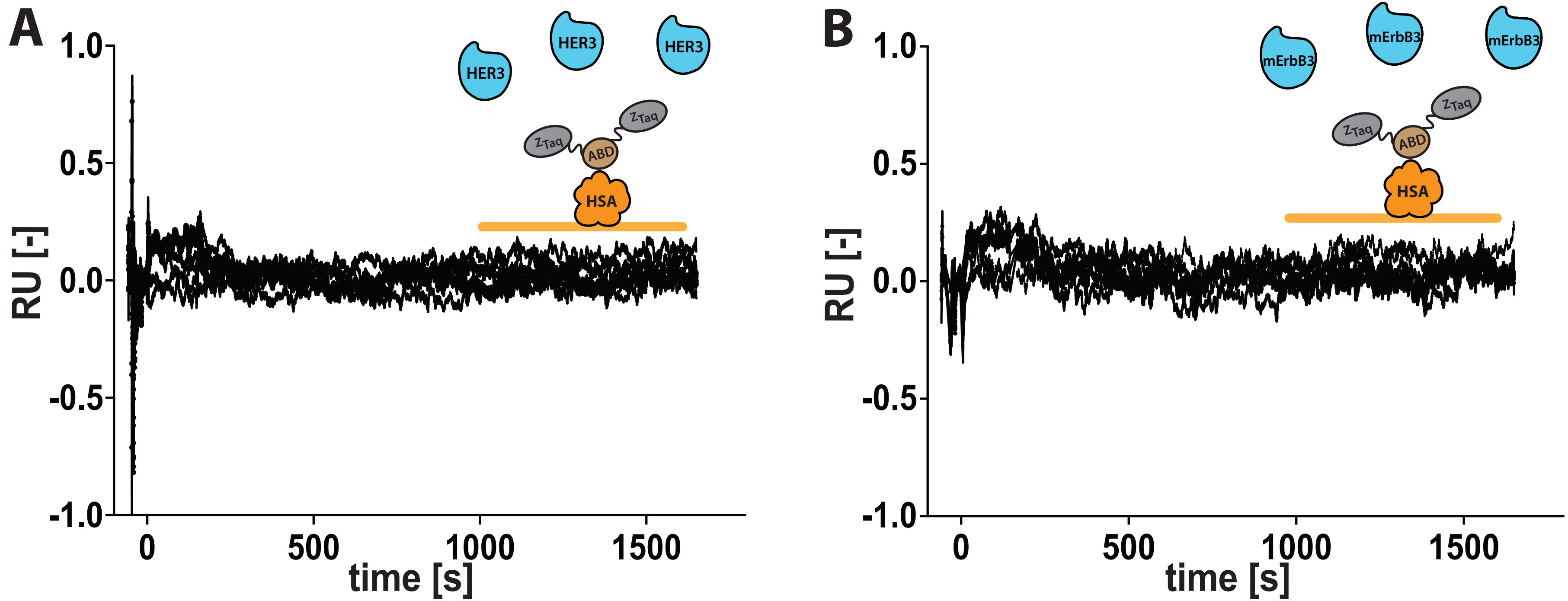

Figure 2S. Supplementary results for the SPR affinity determinations. All analytes were stepwise diluted 1:2 and injected in series over: (**a**) immobilized HSA and TAT captured with subsequent injections of 15.6 to 125 nM of HER3. The dissociation between TAT and HSA has been subtracted, (**b**) immobilized HSA and TAT captured with subsequent injections of 20 to 500 nM of mErbB3. The dissociation between TAT and HSA has been subtracted.
